# Efficacy of Osimertinib in EGFR-Mutated Advanced Non-small-Cell Lung Cancer With Different T790M Status Following Resistance to Prior EGFR-TKIs: A Systematic Review and Meta-analysis

**DOI:** 10.3389/fonc.2022.863666

**Published:** 2022-06-07

**Authors:** Xiao-Fang Yi, Jun Song, Ruo-Lin Gao, Li Sun, Zhi-Xuan Wu, Shu-Ling Zhang, Le-Tian Huang, Jie-Tao Ma, Cheng-Bo Han

**Affiliations:** Department of Oncology, Shengjing Hospital of China Medical University, Shenyang, China

**Keywords:** non-small cell lung cancer, epidermal growth factor receptor, osimertinib, T790M mutation, brain metastases

## Abstract

**Purpose:**

Epidermal growth factor receptor (EGFR) T790M-negative/unknown advanced non-small cell lung cancer (NSCLC) patients lack subsequent approved targeted therapies. This meta-analysis aimed to assess the efficacy of osimertinib in advanced NSCLC patients with different T790M status after resistance to prior first- or second-generation EGFR-tyrosine kinase inhibitors (EGFR-TKIs) and to predict the subgroups that may benefit beside T790M-positive disease.

**Methods:**

PubMed, Embase, Web of Science, and Cochrane Library databases were searched for relevant trials. Meeting abstracts were also reviewed to identify appropriate studies. Studies evaluating the efficacy and/or survival outcomes of osimertinib in patients with different T790M status (positive, negative, or unknown) after resistance to prior first- or second-generation EGFR-TKIs were enrolled, and data were pooled to assess hazard ratios (HRs) or relative risk ratios (RRs) in terms of overall survival (OS), progression-free survival (PFS), and objective response rate (ORR).

**Results:**

A total of 1,313 EGFR-mutated NSCLC patients from 10 retrospective and one prospective studies treated with osimertinib after resistance to first- or second-generation EGFR-TKIs were included. In overall groups, T790M-positive patients showed an improved OS (HR=0.574, p=0.015), PFS (HR = 0.476, p = 0.017), and ORR (RR = 2.025, p = 0.000) compared with T790M-negative patients. In the brain metastases subgroup, no significant difference in OS was observed between T790M-positive and T790M-negative patients (HR = 0.75, p = 0.449) or between T790M-positive and T790M-unknown patients (HR = 0.90, p = 0.673). In the plasma genotyping subgroup, PFS was similar between T790M-positive and T790M-negative patients (HR = 1.033, p = 0.959).

**Conclusion:**

Patients with progressive brain metastases on first- or second-generation EGFR-TKIs can benefit from subsequent osimertinib therapy regardless of T790M status. Patients with plasma T790M-negative status and lack of tissue genotyping should be allowed to receive osimertinib treatment.

## Introduction

Lung cancer is the leading cause of cancer-related mortality, and the most common type is non-small-cell lung cancer (NSCLC), accounting for 85% (1). Because of the high incidence rate and poor prognosis of advanced NSCLC, effective treatment strategies are urgently needed. Activating mutations in the tyrosine kinase domain of epidermal growth factor receptor (EGFR) as one of the significant drivers are mainly found in NSCLC patients; these mutations have motivated the emergency of targeted therapy, which has notably improved the survival of NSCLC patients. For treatment-naive advanced NSCLC patients with EGFR-sensitizing mutations, first-line EGFR-TKIs including first-generation gefitinib, erlotinib, and icotinib, second-generation afatinib and dacomitinib, and third-generation osimertinib and almonertinib have replaced traditional platinum-based chemotherapy as the current therapeutic standard, with a progression-free survival (PFS) range of 9–19.3 months (2–8). Although osimertinib, an irreversible third-generation EGFR-TKI, has been recommended by the National Comprehensive Cancer Network guidelines as a preferred first-line treatment for patients with EGFR-sensitizing mutation advanced NSCLC, first- or second-generation EGFR-TKIs are still an important first-line choice in some parts of the world due to cost and lower overall survival (OS) benefit of osimertinib in subgroups of the Asian population or patients with the 21L858R point mutation compared to first-generation EGFR-TKIs gefitinib and erlotinib observed in the FLAURA study (7).

The most common acquired resistance mechanism to first- or second-generation EGFR-TKIs is a threonine-to-methionine substitution at amino acid position 790 in exon 20 (i.e., T790M mutation), accounting for 49%–73% of the cases of resistance (9–11). Patients with acquired T790M will benefit from subsequent treatment with osimertinib that selectively targets both EGFR-sensitizing mutations and the T790M mutation (12, 13). However, only 50%–60% of resistant patients can undergo tissue rebiopsy to test for T790M (14–16). Plasma circulating tumor DNA (ctDNA), a type of liquid biopsy, is often used as an alternative for genotyping. However, it only exhibits 30%–70% sensitivity for detection of T790M compared with tissue genotyping using next-generation sequencing (NGS) or polymerase chain reaction (PCR)-based detection (17–19). As a result, <30% (14%–27.2%) of patients after resistance to prior EGFR-TKIs can be subsequently treated with osimertinib, and some patients who would likely benefit from osimertinib will go untreated due to a lack of detection or false-negative report of T790M by ctDNA detection (10, 20).

Osimertinib has also been shown to exhibit clinically significant activity for some T790M-negative patients after resistance to first- or second-generation EGFR-TKIs, especially in patients with brain metastasis (BM) (21, 22). Therefore, this meta-analysis aimed to assess the efficacy of osimertinib in advanced NSCLC patients with different T790M status after resistance to prior first- or second-generation EGFR-TKI treatment and to predict the subgroups that may benefit.

## Methods

### Search Strategy

PubMed, Embase, Web of Science, and Cochrane Library databases were searched using the following search terms: (“non-small cell lung cancer” OR “NSCLC”) AND (“osimertinib” OR “AZD9291” OR “third-generation EGFR-TKI”) AND ((“EGFR” AND “mutation”) OR (“epidermal growth factor receptor” AND “mutation”)) to find relevant articles. In addition, abstracts from the American Society of Clinical Oncology (ASCO), European Society of Medical Oncology (ESMO), and World Conference on Lung Cancer were reviewed. Finally, the reference lists of the eligible articles were manually checked to ensure all relevant literature was retrieved. The search end date was October 26, 2021. The article search was performed separately by two investigators.

### Eligibility Criteria

Studies that met the following criteria were included (1): advanced EGFR-mutant NSCLC patients treated with third-generation EGFR-TKIs after resistance to first- or second-generation EGFR-TKIs (2); evaluation of the efficacy and/or survival outcomes of different T790M statuses (positive, negative, or unknown); and (3) outcomes including at least one of the following endpoints, namely, overall survival (OS), PFS, ORR, and duration of response (DOR). The selection of articles was separately performed by two investigators based on a common set of criteria. Differences in opinion were settled through discussion.

### Data Extraction

The extractable data included authors, year of publication, number of patients, gene detection information (T790M positive, negative, or unknown) after resistance to prior-generation EGFR-TKIs, BM status, genotyping sample types, OS, PFS, and hazard ratios (HRs) with 95% confidence interval (CI) for OS and/or PFS, ORR, DOR. Data extraction was performed separately by two investigators.

### Statistical Analysis

All statistical analyses were performed with STATA 14.0 (StataCorp, College Station, TX, USA). The primary endpoints were OS and PFS, and the secondary endpoints were ORR and DOR. The effects of all outcomes were presented with HRs or relative risk ratios (RRs), 95% CIs, and p-values. Subgroup analyses were performed on BM and genotyping samples. HRs and 95% CIs were estimated using the procedures described by Tierney et al. if not reported in a study (23). Kaplan–Meier curve data were recovered *via* Engauge Digitizer version 11.1. This process was repeated two times to reduce variability. The I^2^ statistic was applied to evaluate heterogeneity. The random effect models were chosen if I^2^ was >50% or the p-value was <0.05, implying obvious heterogeneity; otherwise, fixed-effects models were applied. Two‐sided p < 0.05 was considered statistically significant.

## Results

### Characteristics of the Included Studies

**Figure 1:**
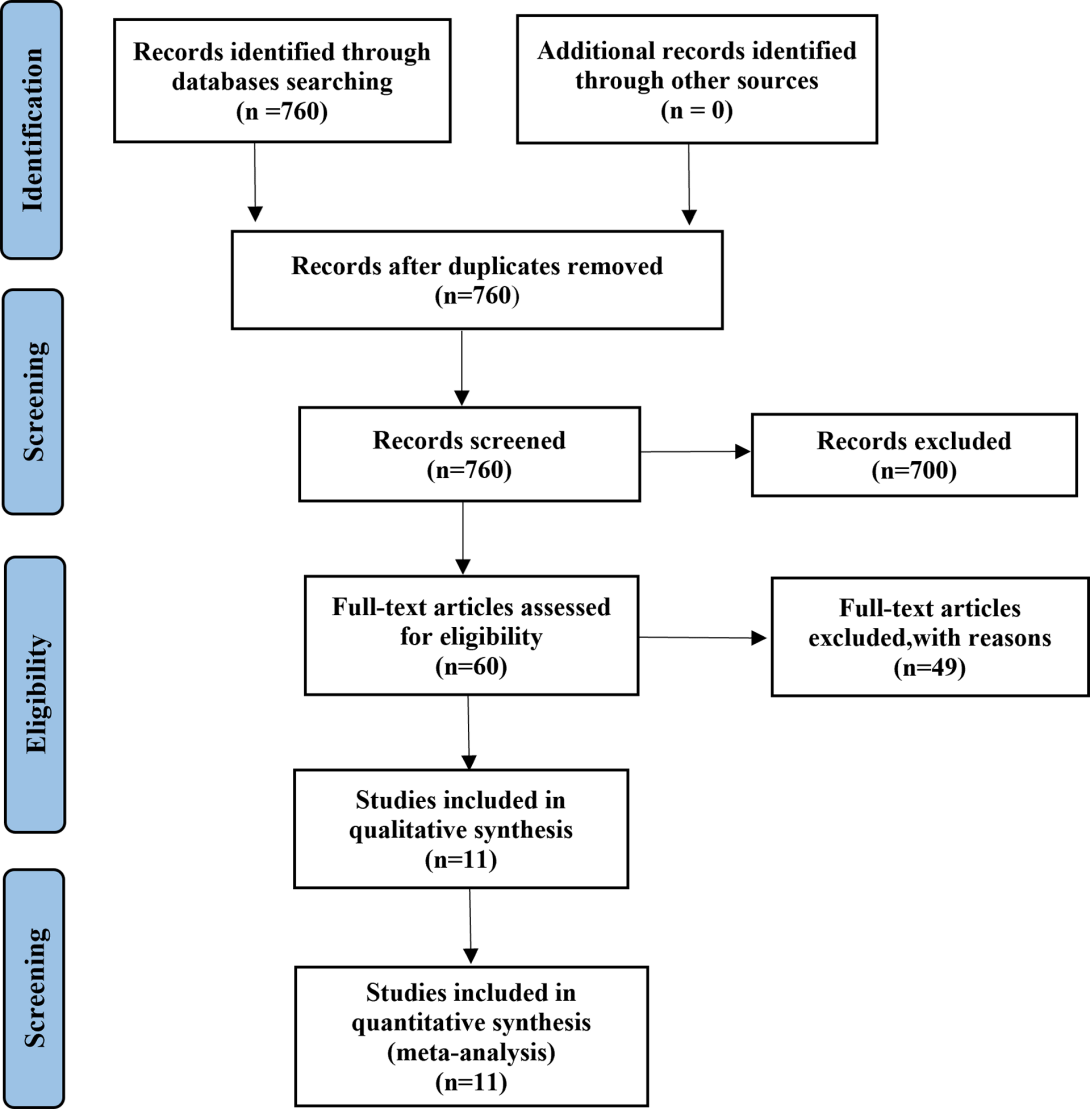
Study selection flow diagram.

**Figure 2:**
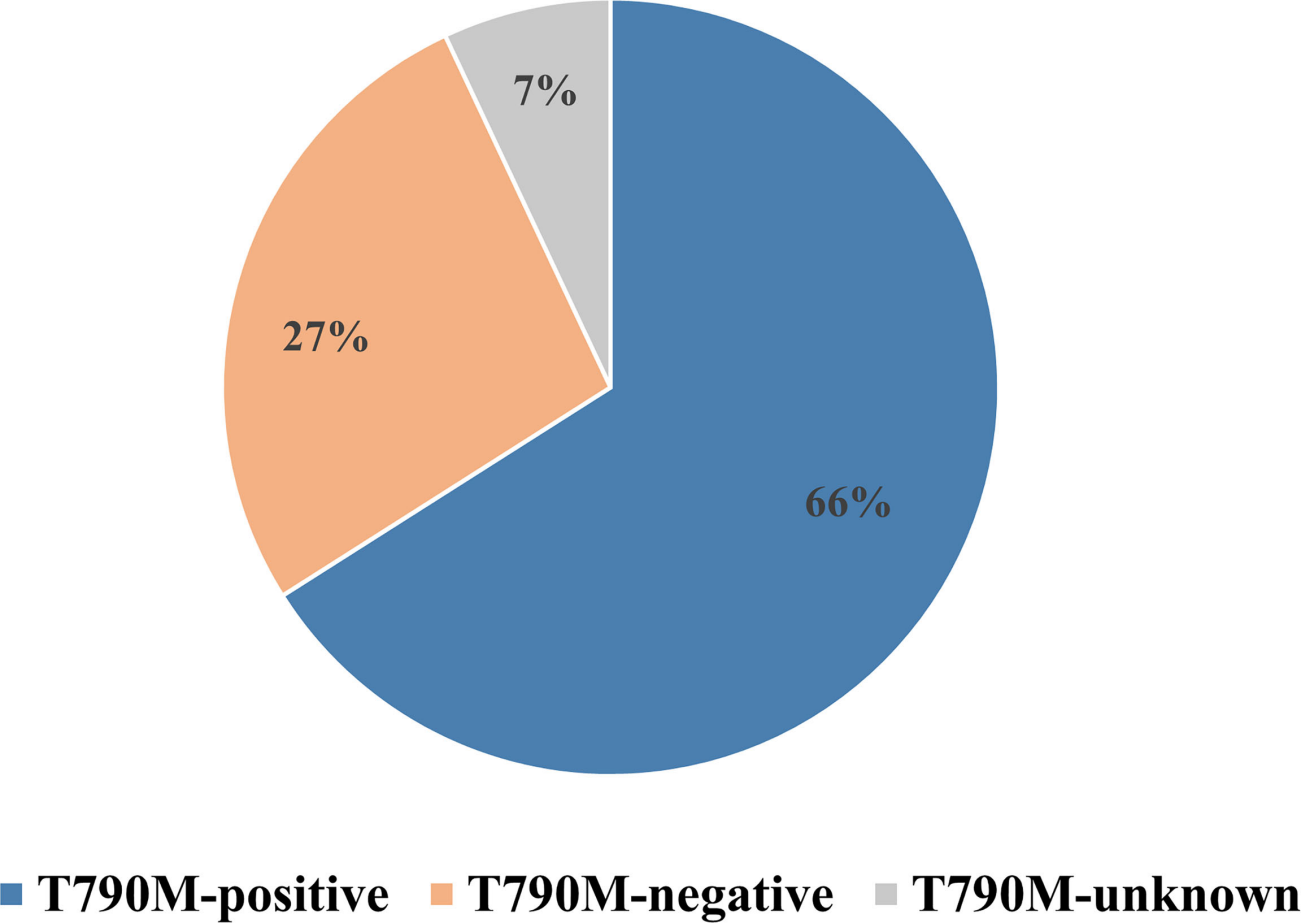
Percentages of T790M-positive, T790M-negative, and T790M-unknown patients in enrolled studies.

A total of 1,313 EGFR-mutated NSCLC patients from 10 retrospective and one prospective study (18, 21, 22, 24–31) treated with osimertinib after resistance to first- or second-generation EGFR-TKIs were included in the meta-analysis **(** **Table 1** **)**. A Preferred Reporting Items for Systematic Reviews and Meta-analyses (PRISMA) flow diagram of the retrieval process is presented in **Figure 1**. Eight studies compared outcomes between T790M-positive and T790M-negative patients, one compared outcomes between T790M-positive and T790M-unknown patients, and two provided survival outcomes of BM subgroups among T790M-positive, T790M-negative, and T790M-unknown patients. The percentages of T790M-positive, T790M-negative, and T790M-unknown patients were 65.80%, 26.70%, and 7.50%, respectively **(** **Figure 2** **)**.

**Table 1:** Study characteristics.

Study(year)	Study arms	No. of patients (n)	No. of BM patients (n)	Genotypingsamples	Study endpoints	Results (T790M+ vs. T790M−/unk)
Xu H^24^ (2021) (26)	T790M+ T790M−	16 24	16 24	Plasma/tissue/CSF	OS/PFS	mOS^a,d^: 11.40 vs. 17.20 mos mPFS^a,d^: 8.80 vs. 10.80 mos mPFS^b^: 8.60 vs. 11.10 mos
Zhang M^22^ (2021) (24)	T790M+ T790M−	28 16	7 16	Plasma/tissue/CSF	OS	mOS^a^: 15.92 vs. 9.00 mos mOS^d^: 22.15 vs. 13.39 mos
Yu X^23^ (2021) (25)	T790M+ T790M− T790M unk	80 15 64	80 15 64	Plasma/tissue	OS	mOS^a,d^: 27.00 vs. 27.00 vs. 27.00 mos
Lee J^25^ (2020) (27)	T790M+ T790M− T790M unk	60 37 13	60 37 13	Plasma/tissue/CSF	OS	mOS^a,d^: 16.70 vs. 18.80 vs. 14.30 mos
Eide IJZ^20^ (2019) (22)	T790M+ T790M−	120 52	– –	Plasma/tissue	OS/PFS/ORR/DoR	mOS^a^: 22.50 vs. 13.40 mos mPFS^a^: 10.80 vs. 5.10 mos ORR^a^: 60 vs. 28% DOR^a^: 11.80 vs. 10.70 mos
Yang JCH^19^ (2019) (21)	T790M+ T790M unk	20 21	20 21	Plasma/tissue	OS/PFS/ORR/DoR	mOS^a,d^: 8.10 vs. 16.60 mos mPFS^a,d^: 8.00 vs. 12.30 mos ORR^a,d^: 45 vs. 38% DOR^a,d^: 7.00 vs. 15.10 mos
Mehlman C^27^ (2019) (29)	T790M+ T790M−	184 35	– –	Plasma/tissue	OS/PFS/ORR	mOS^a^: 27.00 vs. 14.20 mos mPFS^a^: 11.50 vs. 6.00 mos ORR^a^: 54 vs. 40%
Mu Y^26^ (2019) (28)	T790M+ T790M−	77 15	– –	Plasma/tissue	PFS/ORR	mPFS^a^: 8.60 vs. 3.20 mos ORR^a^: 51.40 vs. 26.70%
Saboundji K^28^ (2018) (30)	T790M+ T790M−	13 7	– –	Plasma/tissue	PFS/ORR	mPFS^a^: 49.57 vs. 58.36 mos ORR^a^: 100 vs. 54%
Oxnard GR^16^ (2016) (18)	T790M+ T790M−	164 102	– –	Plasma/tissue	PFS/ORR	mPFS^b^: 9.70 vs. 8.20 mos ORR^b^: 63 vs. 46% mPFS^c^: 9.70 vs. 3.40 mos ORR^c^: 62 vs. 26%
Jänne PA^29^ (2015) (31)	T790M+ T790M−	138 62	– –	Plasma/tissue	PFS/ORR	mPFS^a^: 9.60 vs. 2.80 mos ORR^a^: 61 vs. 21%

BM, brain metastases; CSF, cerebrospinal fluid; DOR, duration of response; mos, months; ORR, objective response rate; OS, overall survival; PFS, progression-free survival; T790M+, T790M-positive; T790M−, T790M-negative; T790M unk, T790M-unknown.

a Overall.

b Plasma genotyping group.

c Tissue genotyping group.

d Brain metastases group.

### Comparison Between T790M-Positive and T790M-Negative Patients

#### Overall Group

**Figure 3:**
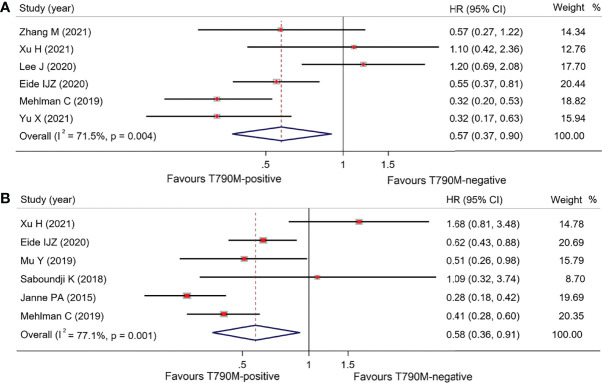
Comparison between T790M-positive and T790M-negative patients of overall group **(A)** and forest plot of OS **(B)**, and forest plot of PFS.

As shown in **Table 2**, overall OS in osimertinib-treated patients was 18.53 months (95% CI, 16.48–20.59) vs. 13.90 months (95% CI, 11.95–15.85) in T790M-positive and T790M-negative groups, respectively, with an HR of 0.57 (95% CI, 0.37–0.90; p = 0.015) (**Figure 3A**). Overall PFS for T790M-positive vs. T790M-negative groups was 9.14 months (95% CI, 8.22–10.06) vs. 3.96 months (95% CI, 3.07–4.85), with an HR of 0.58 (95% CI, 0.36–0.91; p = 0.017) (**Figure 3B**). Overall ORR for T790M-positive vs. T790M-negative groups was 58.41% (95% CI, 52.82–63.99) vs. 24.20% (95% CI, 16.22–32.17), with an RR of 2.03 (95% CI, 1.59–2.58, p < 0.001).

**Table 2:** Pooled results of survival and response rate for the different T790M mutational statuses.

Endpoints	T790M+ vs. T790M−		HR/RR (95% CI)	P value
	No. of studies (patients)	Pooled results (95% CI)		
OS, mos	5 (408) vs. 4 (129)	18.53 (16.48–20.59) vs. 13.90 (11.95–15.85)	0.57 (0.37–0.90)	0.015
PFS, mos	5 (410) vs. 6 (195)	9.14 (8.22–10.06) vs. 3.96 (3.07–4.85)	0.58 (0.36–0.91)	0.017
ORR, %	3 (235) vs. 3 (129)	58.41 (52.82–63.99) vs. 24.20 (16.22–32.17)	2.03 (1.59–2.58)	<0.001
DOR, mos	1 (120) vs. 1 (52)	11.80 (9.85–13.75) vs. 10.70 (5.20–16.20)	NR	<0.001

Abbreviation: 95% CI, 95% confidence interval; DOR, duration of response; HR, hazard ratio; mos, months; NR, not reported; ORR, objective response rate; OS, overall survival; PFS, progression-free survival; RR, relative risk ratio; T790M+, T790M-positive; T790M−, T790M-negative; T790M unk, T790M-unknown.

### Subgroup of Plasma Detection

PFS was not different between plasma detection T790M-positive and T790M-negative subgroups: 9.09 months (95% CI, 8.16–10.02) vs. 9.84 months (95% CI, 8.00–11.69), respectively, with an HR of 1.02 (95% CI, 0.44–2.35) (p = 0.959). ORRs in T790M-positive and T790M-negative subgroups were 63% (95% CI, 55.50–70.50) and 46% (95% CI, 36–56), respectively, with an RR of 1.36 (95% CI, 1.07–1.73; p < 0.001). Tissue genotyping outcomes were extracted from one study with PFS of 9.70 vs. 3.40 months, respectively, in T790M-positive and T790M-negative patients (HR, 0.36; 95% CI, 0.26–0.49) (18). Results are summarized in **Table 3**.

**Table 3:** Pooled results of survival and response rate the T790M-positive and T790M-negative groups with different genotyping samples.

Genotyping method	Endpoints	T790M+ vs. T790M−		HR/RR (95% CI)	p-value
		No. of studies (patients)	Pooled results (95% CI)		
Plasma	PFS, mos	2 (175) vs. 2 (129)	9.09 (8.16–10.02) vs. 9.84 (8.00–11.69)	1.02 (0.44–2.35)	0.959
	ORR, %	1 (164) vs. 1 (102)	3 (55.50–70.50) vs. 46 (36–56)	1.36 (1.07–1.73)	<0.001
Tissue	PFS, mos	1 (173) vs. 1 (58)	9.70 (7.60–11.80) vs. 3.40 (2.30–4.50)	0.36 (0.26–0.49)	<0.001
	ORR, %	1 (173) vs. 1 (58)	62 (54–70) vs. 26 (14–38)	2.39 (1.52–3.76)	<0.001

95% CI, 95% confidence interval; HR, hazard ratio; mos, months; ORR, objective response rate; PFS, progression-free survival; RR, relative risk ratio; T790M+, T790M-positive; T790M−, T790M-negative; T790M unk, T790M-unknown.

### Comparison Among BM Patients With Different T790M Mutation Status

#### T790M-Positive vs. T790M-Negative Groups

**Figure 4:**
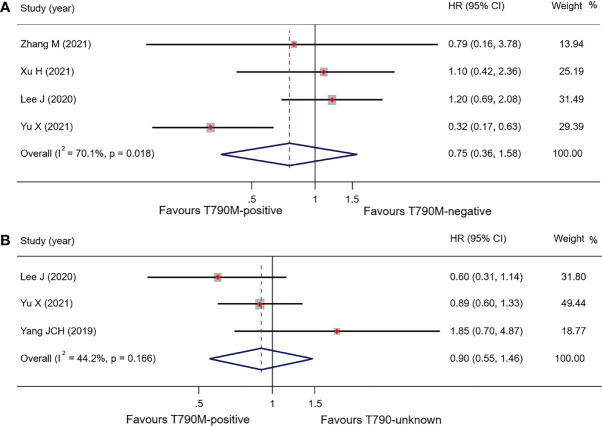
Comparison of BM patients based on T790M mutation status of OS **(A)**, forest plot of comparison of T790M-positive and T790M-negative **(B)**, and forest plot of comparison of T790M-positive and T790M-unknown groups.

Pooled results of the subgroup with regard to BM demonstrated that there was no significant difference in OS between the T790-positive and T790-negative groups. OS in T790M-positive and T790M-negative patients was 16.28 months (95% CI, 13.62–18.94) and 17.50 months (95% CI, 14.61–20.39), respectively, with an HR of 0.75 (95% CI, 0.36–1.58; p = 0.449) (**Figure 4A**). PFS data were only available in one trial: 8.80 vs. 10.80 months, respectively, in T790M-positive and T790M-negative patients (26). These results are summarized in **Table 4**.

**Table 4:** Pooled results of survival and response rate for the different T790M statuses with brain metastases.

Groups	Endpoints	No. of studies (patients)	Pooled results (95% CI)	HR/RR (95% CI)	p-value
T790M+ vs. T790M−	OS, mos	3 (86) vs. 3 (77)	16.28 (13.62–18.94) vs. 17.50 (14.61–20.39)	0.75 (0.36–1.58)	0.449
	PFS, mos	1 (16) vs. 1 (24)	8.80 (7.30–10.30) vs. 10.80 (7.75–13.85)	NR	<0.001
T790M+ vs. T790M unk	OS, mos	3 (160) vs. 3 (98)	20.78 vs. 22.98	0.90 (0.55–1.47)	0.673
	PFS, mos	1 (20) vs. 1 (21)	8 (3–13) vs. 12.30 (5.95–18.65)	NR	<0.001
	ORR, %	1 (20) vs. 1 (21)	45 (22.50–67.50) vs. 38 (16–60)	1.18 (0.57–2.45)	<0.001
T790M+ vs. T790M− vs. T790M unk	OS, mos	2 (140) vs. 2 (52) vs. 2 (77)	22.59 vs. 21.17 vs. 24.86	NR	NR

Abbreviation: 95% CI, 95% confidence interval; HR, hazard ratio; mos, months; NR, not reported; ORR, objective response rate; OS, overall survival; PFS, progression-free survival; RR, relative risk ratio; T790M+, T790M-positive; T790M−, T790M-negative; T790M unk, T790M-unknown.

### T790M-Positive vs. T790M-Unknown Groups

Three studies reported OS in BM patients with T790M-positive and T790-unknown statuses. Pooled OS results in T790M-positive and T790-unknown groups were 20.78 and 22.98 months, respectively (these were calculated using a weighted average of single study medians because of insufficient data of the 95% CI values) (32), with an HR of 0.90 (95% CI, 0.55–1.47; p = 0.673) **(** **Table 4** **;** **Figure 4B** **)**.

### T790M-Positive vs. T790M-Negative vs. T790M-Unknown Groups

A direct comparison of BM patients with the three T790M statuses was also performed in two studies. OS was 22.59, 21.17, and 24.86 months in T790M-positive, T790M-negative, and T790M-unknown groups, respectively; these were calculated using a weighted average of single study medians because of insufficient data of the 95% CI values (32) (**Table 4**).

## Discussion

Patients with advanced NSCLC harboring a secondary EGFR T790M mutation following treatment with first- or second-generation EGFR-TKIs can benefit from subsequent treatment with osimertinib. However, other patients exhibiting resistance with T790M-negative/T790M-unknown statuses lack subsequent approved targeted therapies, and the efficacy of osimertinib in these patients remains unclear. Therefore, it is necessary to explore other subgroups of patients who may benefit from osimertinib treatment to expand its scope of application. Our meta-analysis showed that patients with plasma T790M-negative status or BM patients with T790M-negative or T790M-unknown statuses had similar efficacy to that of T790M-positive patients when treated with osimertinib, suggesting that patients with BM progression with first- or second-generation EGFR-TKIs can benefit from subsequent osimertinib therapy regardless of T790M status, and patients with plasma T790M test-negative status and lack of tissue rebiopsy and genotyping should be allowed to receive osimertinib treatment, especially in the absence of later standard treatment.

Studies have shown that osimertinib can overcome the resistance of acquired T790M mutation, with median PFS of 9.9–12.3 months and ORR of 60–71% (31, 33, 34). A randomized phase III trial, AURA 3, showed that compared with chemotherapy, osimertinib can significantly improve ORR (71% vs. 31%) and PFS (10.1 vs. 4.4 months) in patients with acquired T790M (35). These encouraging results led to the approval of osimertinib as a subsequent treatment for advanced NSCLC patients who developed resistance to prior EGFR-TKIs and acquired a T790M resistance mutation. However, studies have shown that osimertinib also appears to be effective in T790M-negative resistant patients. A study that enrolled 62 T790M-negative patients receiving osimertinib reported a PFS of 2.8 months and an ORR of 21% (31). In a prospective TREM study, 52 EGFR-TKI-resistant patients with T790M-negative status who received osimertinib treatment showed PFS, OS, and ORR of 5.1 months, 13.4 months, and 28%, respectively (22). Furthermore, some retrospective studies have reported that osimertinib had an ORR of 21%–40% and OS of 14–27 months in prior EGFR-TKI-resistant T790M-negative patients (25, 28, 29). This efficacy is similar to the previously reported efficacy of chemotherapy after EGFR-TKI failure. Two studies (AURA3 and IMPRESS) reported PFS of 4.4–5.3 months and ORR of 31.0%–39.5% in patients treated with chemotherapy after resistance to first- or second-generation EGFR-TKIs (35, 36). In our study, the pooled results of osimertinib-treated T790M-negative patients showed similar PFS (3.96 months) and ORR (24.20%) to previous chemotherapy results, indicating that osimertinib may be clinically significant for some patients with a T790M-negative status, although results were not as significant as with T790M-positive patients. However, it is clear that it will be necessary to identify subgroups of these patients that will truly benefit from treatment with osimertinib.

BM progression is a unique disease progression pattern with insufficient response to anti-tumor drugs and poor prognosis because of the active blood–brain barrier (BBB); it accounts for approximately 40% of prior generation EGFR-TKI-resistant metastasis sites (37, 38). In our study, there was no significant OS difference between BM patients with and without T790M, and between those with T790M-positive and T790M-unknown statuses. Furthermore, no significant OS difference was observed in a direct comparison of T790M-positive, T790M-negative, and T790M-unknown patients. These outcomes are generally consistent with the following clinical studies. A retrospective analysis of studies within the AURA series (AURA extension, AURA2, AURA17, and AURA3) exhibited a CNS ORR of 54%–70%, a median CNS PFS of 11.1–11.7 months, and an OS of 18.8 months in T790M-positive patients (33, 34, 39, 40), while some studies also exhibited a CNS PFS of 10.8 months and an OS of 17.2–27 months in T790M-negative patients (24–26). The BLOOM study demonstrated a PFS of 12.3 months and an ORR of 38% in the T790M-unselected population (21). Accordingly, it is worthwhile to discuss whether osimertinib should be used in all patients with progressive BM regardless of T790M status. One of the reasons for the promising efficacy of osimertinib in the CNS may depend on its adequate BBB-penetrating capabilities. The APOLLO and BLOOM studies showed superior BBB penetrations of osimertinib of 31.7% and 16%, respectively (21, 41). However, the BBB penetrations of prior generation EGFR-TKIs were all <6%, with erlotinib at 2.8%–5.1%, gefitinib at 1%–3%, and afatinib at 0.7% (42–45). The insufficient concentration of TKIs in cerebrospinal fluid (CSF), which is less likely to permanently control the dissemination of tumor cells, is crucial in BM after resistance to prior generation EGFR TKIs, apart from the mechanism-induced acquired resistance. Another intriguing circumstance is the mismatching of the T790M mutation detection rate between plasma- or tissue-based genotyping and CSF-based genotyping. A study directly comparing paired plasma and CSF samples in lung adenocarcinoma patients with BM confirmed the lower prevalence of T790M mutation in CSF (3/23) than in plasma (9/23) (46). This result is consistent with other studies reporting a 13%–16% T790M mutation detection rate in CSF, which is significantly lower than the T790M mutation detection rate in plasma of 41%–45% (47, 48). However, one study of 45 EGFR-TKI-treated NSCLC patients with leptomeningeal metastases reported a higher detection rate of the T790M mutation (30.4% vs. 21.7%) and gene copy number variations (CNVs) such as MET (47.8% vs. 0) in CSF than in the plasma, indicating that genetic profiles in CSF may be different from those in plasma, and T790M status in the plasma or primary tumor cannot fully represent the mutation status in CSF (49). In addition, low exposure to first- or second-generation EGFR-TKIs in CSF may also result in “occult” T790M clones within CSF, i.e., low T790M mutation abundance, which may lead to false-negative test results for the T790M mutation. This may be another reason why some patients with BM progression benefit from osimertinib (50). Recently, a study classifying patients into T790M-positive and T790M-negative cohorts based on detection in CSF showed promising efficacy of osimertinib in the T790M-negative cohort with a median intracranial PFS of 7.0 months (51). Thus, plasma and CSF may be complementary for EGFR-TKI resistance patients with BM progression. However, CSF genotyping-based analyses were not included in this meta-analysis for several reasons. First, these data are from retrospective studies with small sample sizes, leading to various biases, such as low statistical power and inflated effect size estimation. Second, because the absolute amount of tumor-derived cell-free DNA in CSF is very low, the method of detecting mutations in CSF is important to the test results. However, techniques used in CSF detection are under exploration with no definitive conclusion. Therefore, osimertinib may be the better choice for patients with BM progression after prior first- or second-generation EGFR-TKIs, regardless of the T790M status.

Tissue genotyping is currently the standard detection approach due to its sensitivity, but is an invasive procedure that may pose danger or cause treatment delays and is often not feasible. For patients inaccessible to tissue biopsy, liquid biopsy, such as plasma genotyping, may be a non-invasive alternative. In the real world, however, approximately 50% of drug-resistant patients underwent tissue rebiopsy, and 20%–50% patients underwent liquid biopsy (20, 52). Previous studies also showed approximately 70% consistency between liquid biopsy- and tissue rebiopsy-based genetic tests in detecting T790M (18, 19). In our meta-analysis, PFS in plasma T790M-positive and T790M-negative patients was 9.09 vs. 9.84 months. PFS provided by one study in tissue T790M-positive vs. tissue T790M-negative patients was 9.7 vs. 3.4 months. There were dramatic differences observed between tissue and plasma genotyping, indicating that there exist sensitivity differences between these methods. The Cobas EGFR Mutation Test v2 for the analysis of T790M in plasma was approved by the US Food and Drug Administration in 2016 because the detection of L858R point mutation and exon 19 deletions in plasma samples with this test method was highly consistent with that in tissue samples (53). Although plasma genotyping has been widely applied in clinical practice, its sensitivity has not been estimated by well-designed, large-scale prospective randomized trials. In terms of the T790M mutation, Arcila et al. had assessed the credibility of plasma genotyping before the emergence of osimertinib (17). Of 64 patients who were confirmed to harbor the T790M mutation with tissue genotyping, 45 were T790M positive with plasma genotyping, including 11 patients who were positive in the second testing, and the overall sensitivity of plasma genotyping was 70%. In the analysis of AURA extension and AURA studies, the sensitivity was 61% and only 51% in the AURA3 study (33, 34, 39). Furthermore, a cross-comparison study of Cobas, Therascreen, ddPCR, and BEAMing provided sensitivities of 41%, 29%, 71%, and 71%, respectively (53). Plasma genotyping has a relatively high positive predictive value, which can avoid biopsies for most patients, but a large proportion of patients with false-negative T790M mutation may miss the chance of osimertinib treatment. For EGFR T790M-negative patients after prior EGFR-TKI therapy, platinum-doublet chemotherapy is considered the standard treatment with a PFS of 4.5–5.4 months and an ORR of 24–30.9% (54, 55). Data on tissue T790M-negative patients treated by osimertinib after failure of prior generation EGFR-TKI treatment are limited; the only study included in this meta-analysis provided a PFS of 3.4 months (95% CI, 2.3–4.5 months) and an ORR of 26% (95% CI, 14–38%) (18). Therefore, osimertinib appears to have similar efficacy compared to chemotherapy but with more manageable toxicity. As a result, for patients in whom tissue genotyping is ultimately unavailable and are plasma T790M-negative, osimertinib is a moderately recommended subsequent line treatment, and for patients who are tissue T790M-negative, osimertinib may also be a choice given that more than a quarter of patients have a response; at the very least, it has certain advantages over chemotherapy.

There are several limitations to this meta-analysis. First, the number of studies and patients included in this pooled analysis is limited. The major reason is that there are few studies assessing the efficacy of osimertinib in advanced NSCLC patients with T790M-negative or T790M-unknown statuses. Second, the included studies are almost all retrospective, with only one prospective study, so selection bias and public bias are difficult to avoid. Third, we failed to further analyze the different detection methods used in the target population after resistance to prior generation EGFR-TKIs, which may have affected the end results. Therefore, larger-scale clinical studies are needed to confirm the efficacy of osimertinib in advanced NSCLC patients with different T790M statuses following resistance to prior generation EGFR-TKIs.

## Conclusion

**Figure 5:**
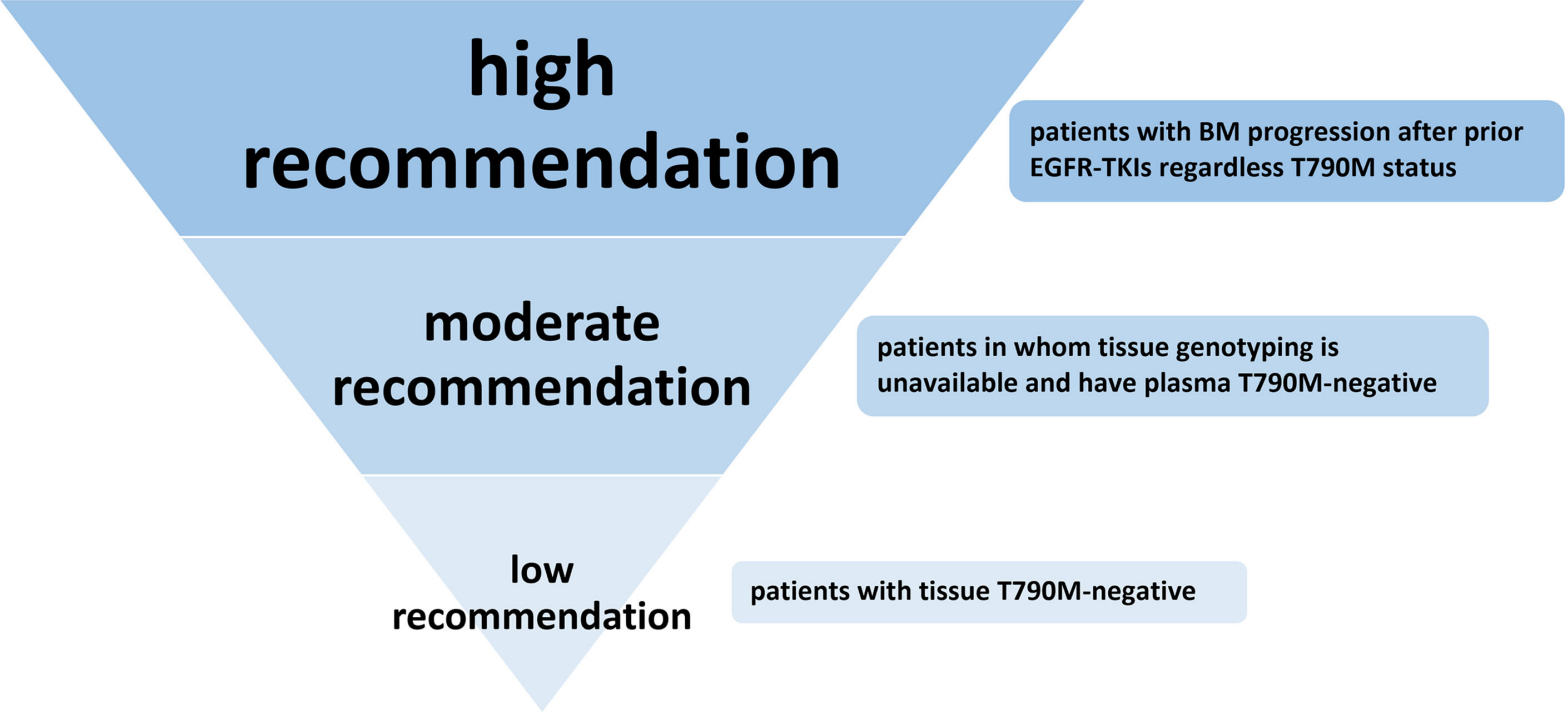
Recommendation level of osimertinib treatment for NSCLC patients with T790M-negative or T790M-unknown status after resistance to first- or second-generation EGFR-TKIs.

Many studies have shown that when off-target (non-EGFR) pathway resistance mechanisms occur, such as MET/HER2 amplification, BRAF mutation, or RET rearrangement, continuously blocking the EGFR pathway with osimertinib in combination with drugs targeting these off-target activating pathway is a promising treatment strategy regardless of the type of EGFR-TKI treatment previously received. Thus, inhibition of the EGFR pathway is important regardless of the cause of EGFR-TKI resistance. This meta-analysis showed that osimertinib has an encouraging efficacy for plasma T790M-negative patients and progressive BM patients regardless of T790M status after resistance to prior generation EGFR-TKIs. Thus, based on the results of this meta-analysis and given the lack of approved effective targeted therapy, we strongly recommend that patients with progressive BM receive osimertinib treatment, even if the T790M test is negative; we moderately recommend osimertinib as a subsequent treatment for advanced NSCLC patients whose tissue rebiopsy is unavailable (T790M-unknown) and plasma T790M test is negative. Finally, for patients who tested negative for T790M by tissue rebiopsy, we only give a low-level recommendation **(** **Figure 5** **)**.

## Data Availability Statement

The original contributions presented in the study are included in the article/supplementary material. Further inquiries can be directed to the corresponding author.

## Author Contribution

X-FY: writing of the original draft. SJ: data extraction and collection. R-LG: data extraction and collection. LS: software. Z-XW: software. S-LZ: formal analysis. L-TH: table editing. C-BH: conceptualization, methodology, and supervision. J-TM: conceptualization, methodology, manuscript review, and revision. All authors contributed to the article and approved the submitted version.

## Funding

This study was supported by grants from the 345 Talent Project of Shengjing Hospital and the Liaoning Province Key Research and Development Plan Projects (No. 2020JH2/10300149).

## Conflict of Interest

The authors declare that the research was conducted in the absence of any commercial or financial relationships that could be construed as a potential conflict of interest.

## Publisher’s Note

All claims expressed in this article are solely those of the authors and do not necessarily represent those of their affiliated organizations, or those of the publisher, the editors and the reviewers. Any product that may be evaluated in this article, or claim that may be made by its manufacturer, is not guaranteed or endorsed by the publisher.
